# Tribological Effects of Water-Based Graphene Lubricants on Graphene Coatings

**DOI:** 10.3390/ma16010197

**Published:** 2022-12-26

**Authors:** Sung-Jun Lee, Yoon-Chul Sohn, Chang-Lae Kim

**Affiliations:** 1 Department of Mechanical Engineering, Chosun University, Gwangju 61452, Republic of Korea; 2 Department of Welding and Joining Science Engineering, Chosun University, Gwangju 61452, Republic of Korea

**Keywords:** water lubrication, friction, wear, graphene

## Abstract

In this study, the friction and wear characteristics of graphene coatings were evaluated using lubricants with various ratios of graphene ink to deionized (DI) water. When dry graphene ink and pure DI water were used as lubricants, the graphene coating initially peeled off, and the friction coefficient rapidly increased to a large value. However, when a lubricant with graphene ink added to DI water was used, a lubricating film was formed on the graphene coating and the friction coefficient was reduced significantly. Under dry and pure DI water conditions, severe wear morphologies were formed on the graphene coating surface, whereas in the case of the lubricant with graphene inks added to DI water, insignificant wear morphologies were formed. When the mixing ratio between DI water and graphene ink was 100:10 and 100:5, the friction coefficient and wear rate were the lowest, respectively. As a result of a long-term experiment in which the sliding cycle was performed for up to 100,000 cycles under the same experimental conditions, the lubricant with a 100:10 mixing ratio showed excellent lubrication properties, confirming that the friction coefficient and wear rate were significantly reduced compared to that of the dry or pure DI water lubrication conditions.

## 1. Introduction

Lubrication has been used in various fields to improve the durability of mechanical parts by reducing friction at the contact surfaces in their contact sliding motion [1,2,3,4]. Oil has excellent lubricating properties and is commonly used as a lubricant. However, the lubricant oil waste is expensive and time consuming to dispose due to its chemical composition, and causes environmental pollution. To solve the problems caused by lubricant waste, eco-friendly lubricants have recently attracted attention [5,6,7]. Studies on water-based clean lubrication are currently being conducted [8,9].

Water has the advantages of being less expensive than oil lubricants, simple to purify after use, and not polluting the environment. However, because water has a lower viscosity than oil and corrodes metal surfaces, its application to mechanical devices made of metallic materials is limited. To solve this problem, studies on creating a water-based lubricant by adding nanoparticles to water, or applying a wear-resistant/corrosion-resistant coating on the surface of the metallic part, have been performed [10,11,12]. It was confirmed through a previous study that when nanoparticles were added, the water lubrication properties were improved by the formation of a lubricating film and the self-lubricating properties of the nanoparticles [13]. However, as the contact sliding state continues, the metal component inside is easily corroded as the surface protective film is worn. It is only by solving the corrosion problem that machinery can be used efficiently and safely. Recently, studies on improving water lubrication properties by developing coatings with excellent corrosion/wear resistance have been reported [14,15].

The possibility of clean lubrication by forming a graphene coating on a metallic material and using only water as a lubricant to improve the friction and wear properties has been suggested [12]. However, when the graphene layers are peeled off by repeated contact and the metal surface is exposed, corrosion and/or wear are intensified. In another case, when an aqueous solution in which graphene particles are dispersed is used as a lubricant, graphene particles are agglomerated on the metal surface by repeated contact to improve the lubrication properties. However, during testing scratches were generated on the metal surface by the counter tip of the tribotester, and as a result it was difficult for the graphene particles to be uniformly formed on the irregularly shaped surface, and the adhesive properties with the surface were not good, so the graphene particles caused abrasive wear.

In this study, a corrosion-resistant coating was applied to the metal surface, and a lubricant in which nanoparticles of the same material as the coating were dispersed in water was simultaneously used to improve the water lubrication properties. Graphene, a low-friction/wear-resistant material, was used both as the lubricant additive and the coating material. By forming a graphene coating on the metal surface and using a lubricant in which graphene, which is the same component as the coating, is dispersed in water, a lubricating film was smoothly formed by the affinity between the graphene coating and the graphene particles to improve the water lubrication properties.

## 2. Materials & Methods

### 2.1. Coating and Lubricants

The graphene solution was prepared by mixing graphene ink (WMCG, MExplorer, Ansan, Korea) with 1.5 wt% of graphene particles and deionized (DI) water in a 1:1 weight ratio and then uniformly dispersed through ultrasonication. A uniformly mixed graphene solution was coated to a thickness of approximately 2 μm using a spray device on the surface of a stainless steel substrate with dimensions of 35 mm × 35 mm and a thickness of 1 mm and heated to 200 °C. It was heated on a hot plate at 200 °C for approximately 30 min so that the graphene coating was firmly adhered on the substrate surface. Lubricants were prepared with various mixing ratios of DI water and graphene ink. Table 1 lists the lubricants and coating conditions used in this study. The mixing wt% ratio of the graphene lubricant was selected as DI water (100): nanoparticles (0, 1, 5, 10, and 20). After the graphene ink was added to the DI water, the aqueous solution was mixed for approximately 1 h using a magnetic stirrer. It was then uniformly dispersed by sonication for 10 min.

### 2.2. XRD and Raman Analysis

The structure and defects of the graphene coating were analyzed using X-ray diffraction (XRD, EMPyrean, PANalytical, Malvern, UK) and Raman spectroscopy (DRX, Thermo Scientific, Waltham, MA, USA). XRD analyzes the crystallinity and microstructure using the principle that diffraction angles differ according to the crystallinity. The measurement range of the diffraction angle was 5–50°, and the peak value was obtained according to the diffraction angle. Raman spectroscopy is an analytical method used to obtain the molecular structure of a substance by measuring the energy is lost through phonons emitted during light scattering that occurs when matter is irradiated by light. Raman analysis was performed after observing the nanoparticles on the substrate under a microscope. The laser wavelength of the Raman device was 532 nm, and the spectrum was measured in the range of 500–4000 nm with a power of 6 mW.

### 2.3. Tribo-Test Condition

A tribotester with a reciprocating sliding motion (RFW 160, NEOPLUS, Co., Ltd., Daejeon, Korea) was used to evaluate the friction and wear characteristics of the graphene coating according to the amount of graphene ink added to the lubricant. The tribotester converts the rotational motion of the motor into linear motion through a cam connected to the shaft to enable repeated sliding motion. The tribo-test conditions are listed in Table 2. For the counter tip, a stainless steel sphere with a diameter of 1 mm was selected. After the graphene-coated specimen was fixed on the reciprocating stage, the counter tip was brought into contact with the specimen surface in a leveled state through movement of the weight balance. After a 200 mN vertical load was applied, the counter tip was slid on the specimen surface with a sliding stroke of 2 mm at a sliding speed of 16 mm/s for 10,000 cycles. Except for the lubrication condition, all other experimental conditions were the same, and the reliability of the experimental results was secured by repeating the experiment three or more times. After the experiment, 3D laser scanning confocal microscopy (3D LSCM, VK-X200, KEYENCE, Osaka, Japan) and scanning electron microscopy (SEM, JEOL, Tokyo, Japan) were used to analyze the morphology of the wear track to determine the wear depth and width.

## 3. Results & Discussion

### 3.1. X-ray Diffraction & Raman Spectroscopy

**Figure 1:**
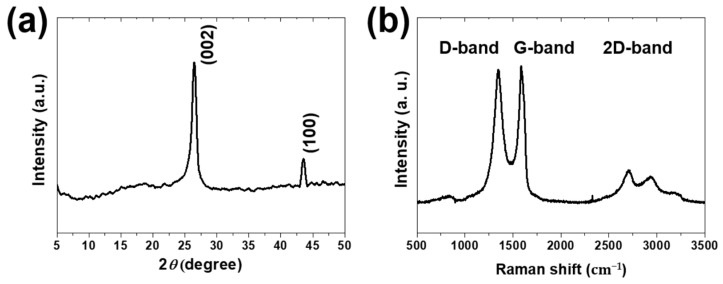
(**a**) XRD pattern of graphene and (**b**) Raman spectrum of the graphene coating.

The multilayer structure and defects of the graphene coating were analyzed using XRD and Raman spectroscopy. The XRD pattern of graphene is shown in Figure 1a. The characteristic values obtained from the XRD data are summarized in Table 3 [16,17,18]. X-ray diffraction peaks were observed at 26° and 43°, and the interplanar spacings were 3.37 Å and 2.08 Å, respectively. For each peak, the crystal planes of (002) and (100) have been shown in previous studies. It shows the highest peak at 26°, indicating the formation of a layered graphene structure with an interlayer distance of 0.3 nm. In the XRD analysis of graphene, a peak near 11° was observed when graphene was oxidized and disappeared after a reduction reaction [19,20,21]. Therefore, it was concluded that graphene was reduced because the peak at approximately 11° was not observed. Figure 1b shows the Raman spectrum of the graphene coating. Graphene, composed of carbon atoms, forms a hexagonal lattice by sp_2_ bonding, so the Raman spectrum obtained is similar. Three distinct peaks were observed near 1350 cm^−1^, 1585 cm^−1^, and 2700 cm^−1^ corresponding to the D and G bands. The peak near 1585 cm^−1^ is the G band owing to sp_2_ bonding, and the peak near 2700 cm^−1^ is the 2D band owing to multiple scattering [22,23]. The D band observed near 1350 cm^−1^ appears because of the symmetry of the vibrational motion due to defects in the crystal, and it is difficult to observe in a perfect lattice structure [24]. Therefore, when there are many defects, the peak of the D band appears larger than that of the G band in some cases; therefore, the ratio of the D and G peaks is used as an indicator of defects in graphene [25]. The I_D_/I_G_ value of graphene used in this study was 0.92, showing a trend similar to that of previous studies [26,27,28].

### 3.2. Tribological Characteristics

**Figure 2:**
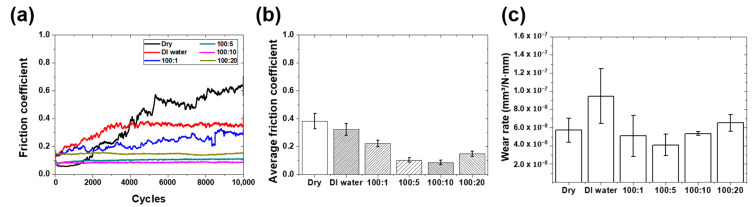
Friction and wear characteristics of graphene-coated stainless steel according to the mixing ratio of DI water: graphene ink (**a**) friction coefficient history, (**b**) average friction coefficient, and (**c**) wear rate.

The lubrication properties of lubricants in which the graphene ink was mixed with DI water at various ratios for graphene coating were evaluated. Figure 2a shows the change in the friction coefficient with an increase in the number of sliding cycles performed in the friction tests. The tests were performed for 10,000 cycles using lubricants with various mixing ratios. Figure 2b shows the average friction coefficient values according to lubrication conditions. The average friction coefficient of the graphene coating was measured at 0.38 in dry conditions, and 0.09–0.32 in lubricated conditions.

Under dry conditions, the graphene coating showed a low friction coefficient of 0.1 at the beginning, but the friction coefficient increased rapidly after 1000 cycles. After 1000 cycles, wear appears to have occurred in the graphene coating, and it is believed that the wear accelerated rapidly and the graphene coating peeled off [29]. Moreover, after 5000 cycles, the friction coefficient increased to approximately 0.6, and after 8000 cycles, the friction coefficient increased to approximately 0.7. This friction coefficient value was determined because of direct contact between the stainless-steel substrate and the counter tip of the tribotester after the coating layer was removed. The mechanical strength of graphene is 200 times greater than that of iron, and its durability is excellent [30]. However, oxidized/reduced multilayered graphene-like graphite is easily destroyed by an external force because it is bonded by the attractive force between graphene layers and has a low mechanical strength compared to that of pure single-layer graphene [31,32].

When DI water was used as the lubricant, the average friction coefficient was measured to be 0.32, which was slightly lower than that under dry conditions. The initial friction coefficient was approximately 0.15, which was slightly higher than that under dry conditions, and it increased rapidly from the beginning. The friction coefficient increased up to 3000 cycles and then remained constant at approximately 0.37 from 3000 cycles to 10,000 cycles. Since graphene was well dispersed in DI water, there is a possibility that graphene particles may have come off from the graphene coating under DI water lubrication conditions. The tests indicate that the lubrication effect on the graphene coating by pure DI water is insufficient, and the graphene particles are easily separated, resulting in a higher friction coefficient compared to the dry condition. However, because the graphene particles generated during DI water lubrication were formed into a new lubricating film by repeated contact sliding motion, it was considered that the friction coefficient did not increase any further and remained constant after 3000 cycles.

When lubricants mixed with graphene ink and DI water in various ratios were used, the friction coefficients were measured to be 0.09–0.22. In the case of using a mixed lubricant of 100:10 ratio, the friction coefficient was 0.09, which was the lowest, decreasing by 76% and 72%, respectively, compared to those of dry and pure DI water conditions. When DI water containing graphene ink was used as a lubricant, except for the lubricant with a mixing ratio of 100:1, the friction coefficient value was maintained low without significant change in the friction coefficient. When the mixing amount of the graphene ink is small, it is difficult to expect low friction characteristics due to the non-uniformity of the graphene particles. For that reason, the relatively low friction coefficient is maintained by the formation of graphene particles in the mixed lubricant to form a thick lubricating film. As the mixing amount of the graphene ink increased, the friction coefficient showed a tendency to decrease; however, when a lubricant with a mixing ratio of 100:20 was used, the friction coefficient increased. In the case of a lubricant with a mixing ratio of 100:20, the graphene lubricating film with an irregular structure was formed by excessive graphene particles, and the friction coefficient increased because the counter tip of the tribotester was caught by the agglomerated graphene particles formed on the surface of the specimen [33,34].

After the friction test was completed, the wear width and depth were measured using a 3D laser scanning confocal microscope and the wear rate was calculated using the following formula [35]:

Wear rate = (W.V.)/(L × D) (1)

The wear rate [mm^3^/N·mm] is the wear volume (W.V.) [mm^3^] divided by the normal load (L) [N] and total sliding distance (D) [mm]. The wear rate of each lubricant is shown in Figure 2c. The wear rate in the dry condition was 5.73 × 10^−8^ mm^3^/N·mm, and the wear rate in the lubricated condition was calculated as 9.47 × 10^−8^–4.1 × 10^−8^ mm^3^/N·mm. When pure DI water was used as a lubricant, the wear rate was the highest at 9.47 × 10^−8^ mm^3^/N·mm. Since water dispersion graphene exhibits a property of being well dispersed in water, we found that the graphene particles are easily peeled from the graphene coating due to their hydrophilicity under DI water lubrication conditions. Although the friction coefficient is reduced because the exfoliated graphene particles form a new lubricating film, it is considered that the wear rate is high because some of the particles already separated from the coating layer function as a lubricating film and the remaining particles are accumulated by repeated sliding motion. When the DI water lubricant mixed with graphene ink is applied, it seems that the friction coefficient and wear rate are reduced because the graphene particles present in the lubricant form a lubricating film on the surface to some extent from the beginning of the sliding motion. When a mixed lubricant with a 100:5 ratio was used, the wear rate was 4.1 × 10^−8^ mm^3^/N·mm, which decreased by 28% and 57% compared to that of the dry and DI water lubrication conditions, respectively, and it was the lowest. The wear rate when using a mixed lubricant with a 100:20 ratio, which had the highest mixing ratio of graphene ink, was higher than that in the case of dry lubrication. In the case of a lubricant in which more than a certain amount of graphene particles is mixed, the graphene particles tend to agglomerate and function as abrasive particles, which intensifies wear.

**Figure 3:**
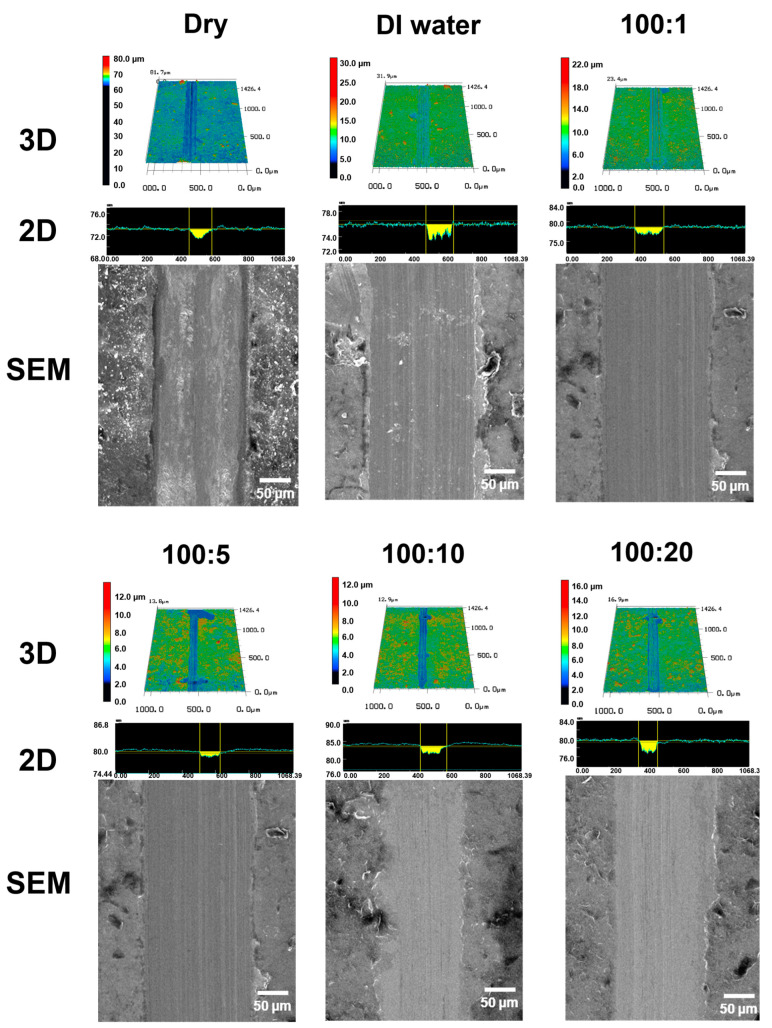
3DLaser scanning confocal microscope and SEM images of wear tracks with respect to the lubrication conditions under 200 mN after 10,000 cycles.

Figure 3 shows the 3D LSCM and SEM images of the wear tracks formed on the surfaces of the specimens after the friction tests. Highly rough surfaces were formed inside the wear tracks under dry and pure DI water lubricated conditions. It can be seen that the graphene coating is damaged by repeated sliding motion and the wear is aggravated by insufficient formation of the lubricating film. In the case of lubricants mixed with graphene and DI water, smoother wear tracks were formed than in the cases of dry and pure DI water lubrication conditions, and slight scratch marks were formed on the surfaces of the wear tracks.

**Figure 4:**
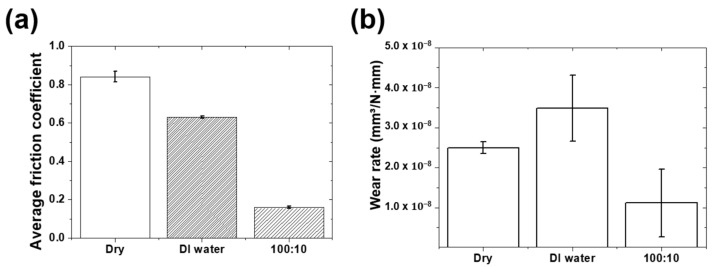
Friction and wear characteristics of graphene coated stainless steel under 200 mN normal loads during 100,000 cycles. (**a**) average friction coefficient; (**b**) wear rate.

An additional friction test was performed for 100,000 cycles to evaluate the persistence of the lubricating properties for the mixed lubricant with a ratio of 100:10 with the lowest friction coefficient. Dry and DI water lubrication conditions were set as a comparison group, and friction tests were conducted under the same conditions for a long time, and the friction coefficients according to each lubrication condition were compared, as shown in Figure 4a. Under dry conditions, the friction coefficient for 100,000 cycles was 0.84, which was more than twice than that of 10,000 cycles, indicating a significant increase. As the number of sliding cycles increased, the friction coefficient increased significantly owing to the rough surface caused by the intensification of the wear of the graphene coating. That is, under dry conditions, it is judged that the graphene coating alone is insufficient to serve as a solid lubricant for long-time contact sliding, and the coating surface is completely damaged. Under pure DI water lubrication conditions, the friction coefficient for 100,000 cycles was 0.63, which is almost double than that for 10,000 cycles. Even if pure DI water was used as a lubricant, there was a limit to preventing the breakage of the graphene coating as the number of sliding cycles increased, and it is considered that the friction coefficient increased owing to the rough surface of the wear track. For a mixed lubricant with a ratio of 100:10, the average friction coefficient was 0.16 for 100,000 cycles of sliding motion, maintaining the lowest friction coefficient. It is considered that a lubricating film was formed by the graphene particles mixed in the DI water, thus protecting the surface from contact sliding for a long time.

As shown in Figure 4b, the wear rate due to sliding for a lengthy period of time (100,000 cycles) in the dry condition was 2.5 × 10^−8^ mm^3^/N·mm, which was more than double than that in the case of 10,000 cycles under the same conditions. This indicates that a larger amount of wear was generated by the long sliding motion, but the rate at which the amount of wear increased decreased as the number of sliding cycles increased. That is, the increase in the wear rate is reduced, so that the wear rate during the entire sliding cycle is greatly reduced. It is thought that this is because the graphene coating is worn and the wear rate increases as the graphene particles are separated from the coating surface, and the particles form a lubricating film on the surface and prevent the expansion of wear to some extent. It is expected that the process of forming the lubricating film again after the newly formed lubricating film breaks again and falls out as wear particles are repeated. However, when sliding motion was performed for a long time under pure DI water lubrication conditions, the wear rate showed the highest value of 3.49 × 10^−8^ mm^3^/N·mm, and it decreased by 2.7 times compared to that of the sliding motion of 10,000 cycles. The increase in the rate of wear decreased with an increase in the sliding cycles. This is because the graphene particles of the coating fell off after pure DI water was applied as a lubricant, causing continuous wear; however, the graphene particles that fell off were again partially formed into a lubricating film. Even in this case, the formation and destruction of the graphene lubricating film may have occurred repeatedly. In contrast, for the lubricant mixed at a ratio of 100:10, the wear rate for long-time sliding motion was 1.11 × 10^−8^ mm^3^/N·mm, showing the lowest value, and it was reduced by nearly five times compared with that of the sliding motion of 10,000 cycles. This is because the increase rate of wear owing to the increase in the sliding cycle is the lowest, and it is considered that the graphene particles mixed in the DI water form a lubricating film that protects the surface and reduces friction. The wear rates for the DI water-based graphene lubricant were reduced by approximately 56% and 68%, respectively, compared to those under dry and pure DI water lubrication conditions.

**Figure 5:**
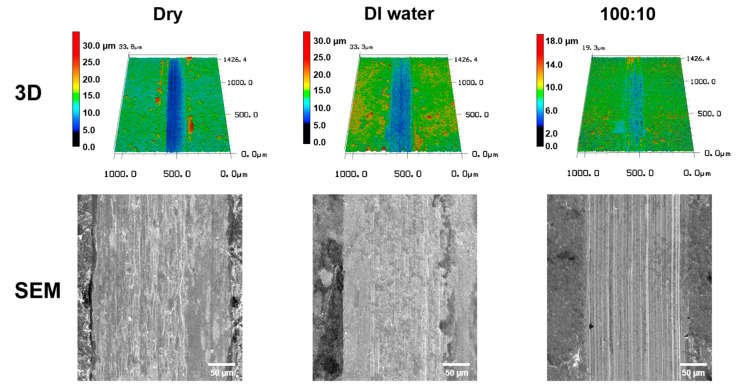
Laser scanning confocal microscope and SEM images of wear track under 200 mN during 100,000 cycles.

**Figure 6:**
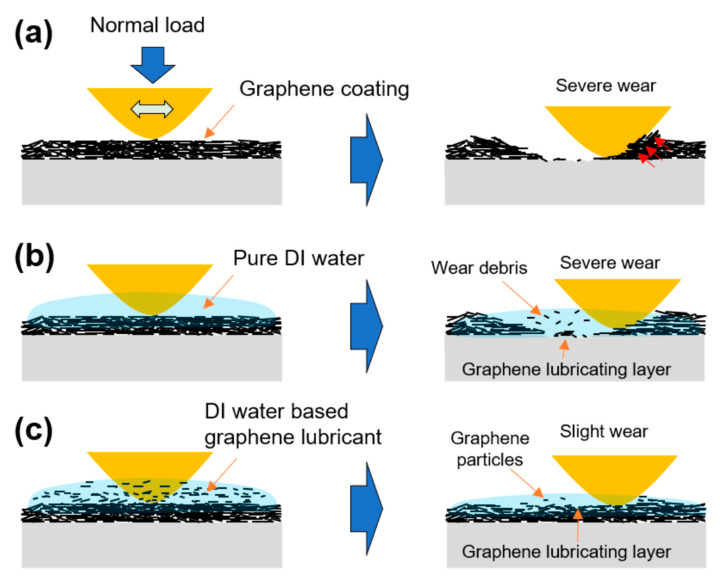
Schematic design of wear mechanism of graphene coating under (**a**) dry, (**b**) pure DI water and (**c**) DI water-based graphene lubricant conditions.

Figure 5 shows the wear tracks formed on the graphene-coated surface after sliding motion for 100,000 cycles. Under dry and pure DI water lubrication conditions, the surface inside the wear track was very rough, whereas when the DI water-based graphene lubricant was used, the wear track formed was the smallest and only scratches in the sliding direction were generated. The graphene particles in the graphene solution used to form the graphene-coated specimen by the spray-coating method were uniformly applied to the surface. In contrast, under dry and pure DI water lubrication conditions, the graphene particles separated from the graphene coating by repeated contact sliding with the counter tip accumulated on the surface of the wear track and formed an intermittent lubricating film. The wear mechanism of the graphene coating under dry, pure DI water, and mixed lubrication conditions are shown in Figure 6.

**Figure 7:**
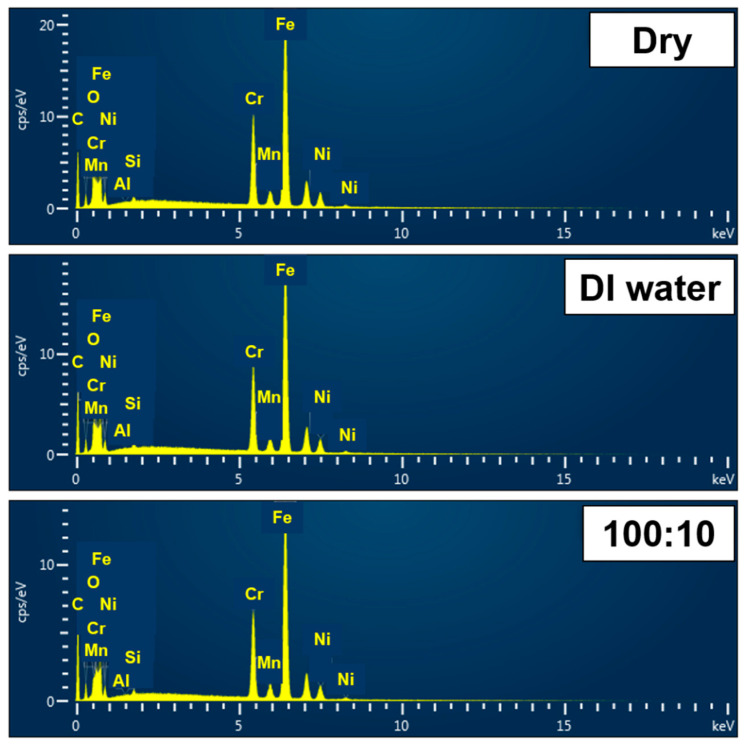
Results of EDS analysis on wear track surface after tribo-test during 100,000 cycles.

EDS analysis was used to determine the degree of breakage of the worn surface, and the results are shown in Figure 7. Under dry conditions, 20% carbon and 18% oxygen were measured in the wear track of the graphene coating. This result proves that the carbon-based graphene coating layer is almost exfoliated, and the oxygen content is relatively high compared with that of other lubrication conditions. As the surface of the stainless-steel plate was damaged and exposed to the atmosphere, corrosion was expected to progress in the wear track. Under pure DI water lubrication, the analysis results were like those obtained under dry conditions with 24% carbon and 10% oxygen. Severe abrasion occurred to the extent that the graphene coating was completely removed, and it was considered that the wear rate was high accordingly. In the case of DI water-based graphene lubricant with a ratio of 100:10, 30% carbon and 2.74% oxygen were measured in the wear track of the graphene coating, indicating that the oxygen content was relatively small. This proves that that the graphene coating layer remains on the wear track surface, and it is expected that corrosion is relatively minor compared to that of other lubricating conditions as the lowest oxygen content was measured.

## 4. Conclusions

In this study, DI water-based graphene lubricants were prepared by adding graphene ink to pure DI water to improve the water-based lubrication properties of the graphene coatings. In the friction test under dry conditions, the friction coefficient was high because wear debris was generated on the graphene-coated surface by repeated sliding motion. When pure DI water was used as a lubricant, the friction coefficient was reduced, but the graphene layers were easily peeled off, and a non-uniform lubricating film was formed, which intensified the wear. In contrast, in the case of the DI water-based graphene lubricants, both the friction coefficient and wear rate were significantly reduced. The friction coefficient and wear rate were the lowest when the mixing ratios were 100:10 and 100:5, respectively. Graphene particles dispersed in DI water formed a lubricating film that exhibits friction-reducing properties and surface protection. Even in the case of long-time sliding motion, only in the case of DI water-based graphene lubricants, the graphene coating was not completely damaged, and the slightest wear occurred. The results of this study will be helpful for studies to improve the water lubrication properties of metal substrates and may suggest the applicability of water-based eco-friendly lubricants by optimizing the amount of graphene added.

## Figures and Tables

**Table 1:** Coating fabrication and lubrication conditions.

Lubrication Condition Mixing Ratio (DI Water: Graphene ink)	Substrate	Coating Method	Coating Condition	Heating Condition
Dry	Stainless steel	Spray coating	○ Thickness: 2 µm ○ Temperature: 200 °C	○ Temperature: 200 °C ○ Time: 30 min
100:0				
100:1				
100:5				
100:10				
100:20				

**Table 2:** Tribo-test conditions.

Tribo-Test (Reciprocating Type)	
Tip Material (Diameter)	Stainless Steel Ball (D: 1 mm)
Normal load	200 mN
Sliding speed	16 mm/s
Sliding stroke	2 mm
Sliding cycle	10,000/100,000 cycles
Lubrication condition	Dry/DI water/0.1, 1, 10, 100 wt% graphene-DI water based lubricants

**Table 3:** Results of XRD analysis of graphene-coated stainless steel.

2θ (°)	d (Å)	hkl	FWHM (°)	Crystallite Size (nm)
26.44	3.36871	002	0.5519	11
43.49	2.07908	100	0.3735	16.8

## Data Availability

Data is available on request from the corresponding author.
